# A case of groove pancreatic carcinoma presenting with rapidly progressive ascending colon stenosis: a rare case report

**DOI:** 10.1097/RC9.0000000000000853

**Published:** 2026-08-14

**Authors:** Shohei Shiozaki, Manabu Kurayoshi, Kosuke Ono, Masahiro Nakahara

**Affiliations:** Department of Gastroenterological and Endoscopic Surgery, JA Onomichi General Hospital, Onomichi, Hiroshima, Japan

**Keywords:** ascending colon stenosis, case report, diagnostic difficulty, groove pancreatic carcinoma, retroperitoneal invasion

## Abstract

**Introduction:**

Groove pancreatic carcinoma is a rare pancreatic cancer arising in the space between the pancreatic head, the duodenum, and the common bile duct. Owing to its unusual growth pattern and its resemblance to inflammatory conditions such as groove pancreatitis, preoperative diagnosis remains challenging.

**Presentation of the case:**

An 80-year-old man presented with abdominal pain and progressive stenosis of the ascending colon. Endoscopy demonstrated edematous narrowing of the duodenum and ascending colon without mucosal abnormalities. Cross-sectional imaging showed inflammatory changes around the pancreatic groove, but no definite mass lesion. Two months later, bowel obstruction developed due to the rapid progression of colonic stenosis, and a right hemicolectomy was performed. Histopathology demonstrated metastatic poorly differentiated adenocarcinoma involving the colonic wall. Postoperative positron emission tomography-computed tomography (PET-CT) revealed a retroperitoneal mass; however, the primary site remained unclear. Three months later, the disease progressed rapidly with multiple hepatic metastases. A pathological autopsy revealed a tumor in the pancreatic groove region with extensive retroperitoneal invasion, leading to the final diagnosis of groove pancreatic carcinoma.

**Discussion:**

Groove pancreatic carcinoma commonly presents with duodenal stenosis and is frequently difficult to distinguish from inflammatory conditions. Direct invasion of the colon is extremely rare. In this case, retroperitoneal tumor spread from the pancreatic groove resulted in rapidly progressive ascending colon stenosis, which preceded the diagnosis of the primary tumor.

**Conclusion:**

This case highlights the diagnostic difficulty and unusual clinical presentation of groove pancreatic carcinoma. Rapidly progressive colonic stenosis adjacent to the pancreatic groove should raise suspicion for this rare malignancy, even in the absence of typical pancreatic findings.

## Introduction

The pancreatic groove region is surrounded by the pancreatic head, the descending duodenal limb, and the lower common bile duct. Gabata *et al*[1] reported that pancreatic head cancer infiltrating the groove region should be referred to as “groove pancreatic carcinoma.” Groove pancreatic cancer requires careful differentiation from groove pancreatitis, making it difficult to diagnose preoperatively; pathologically, it resembles pancreatic ductal adenocarcinoma. Compared with typical pancreatic head cancer, it more commonly causes duodenal stenosis and gastric emptying disorders but rarely involves the colon or presents with colonic symptoms[2]. Herein, we report a case of groove pancreatic cancer with ascending colon stenosis that progressed rapidly and was difficult to diagnose preoperatively. This report was prepared according to the SCARE guidelines[3].

HIGHLIGHTS

- Groove pancreatic carcinoma is a rare pancreatic malignancy that is often difficult to diagnose preoperatively.
- This case presented with rapidly progressive stenosis of the ascending colon without mucosal abnormalities, which is an extremely unusual manifestation.
- Retroperitoneal tumor spread from the pancreatic groove directly invaded the ascending colon, causing bowel obstruction.
- The primary tumor could not be identified during life and was definitively diagnosed by pathological autopsy.
- Unexplained colonic stenosis near the pancreatic groove should prompt consideration of groove pancreatic carcinoma.

## Case presentation

The patient was an 80-year-old man who presented with abdominal pain. He was referred to our department of gastroenterology with complaints of fever, abdominal pain, and jaundice. His past medical history included alcoholic liver disease, chronic kidney disease, and diabetes mellitus. The abdomen was distended but soft, with no marked tenderness.

**Figure 1.**
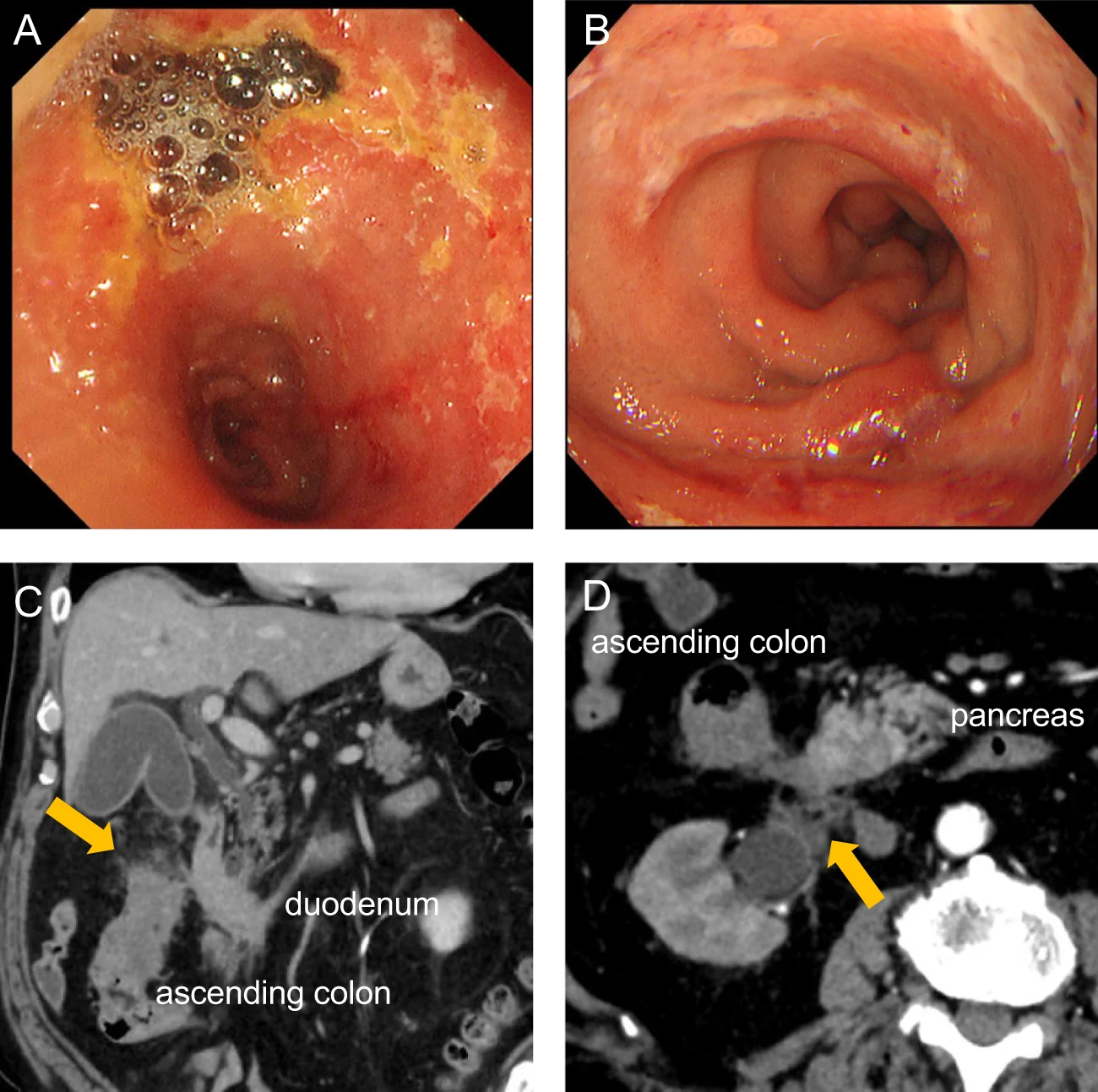
Initial upper gastrointestinal endoscopy and contrast-enhanced CT findings. (a, b) Upper gastrointestinal endoscopy revealed edematous narrowing extending from the duodenal bulb to the descending portion of the duodenum, without any obvious mucosal tumor lesions. (c) Contrast-enhanced computed tomography (CT) demonstrated increased fat attenuation surrounding the duodenum, ascending colon, and the anterior renal fascia (arrow). (d) Dilatation of the right ureter and right renal pelvis was observed, with a transition point located adjacent to the right ureter (arrow).

Blood tests revealed elevated hepatobiliary enzyme levels, particularly biliary enzymes, along with renal dysfunction. Tumor markers, including carcinoembryonic antigen and carbohydrate antigen 19-9, were within normal limits. Upper gastrointestinal endoscopy revealed edematous narrowing from the duodenal bulb to the descending duodenum, without obvious mucosal tumor lesions (Fig. 1a and b). Endoscopic ultrasonography showed no definitive malignant mass and no stenosis or dilation of the common bile duct or main pancreatic duct. No endoscopic retrograde cholangiopancreatography (ERCP) was performed because mucosal edema narrowed the bile duct, making cannulation difficult. Lower gastrointestinal endoscopy revealed edematous narrowing of the ascending colon; however, no obvious mucosal abnormalities were observed. The endoscope passed through the region without difficulty, indicating no evidence of obstructive stenosis. Contrast-enhanced CT revealed increased fat attenuation surrounding the duodenum, ascending colon, and anterior renal fascia (Fig. 1c). Dilatation of the right ureter and right renal pelvis was observed, with a transition point near the right ureter (Fig. 1d). Magnetic resonance cholangiopancreatography (MRCP) revealed no evidence of gallstones, common bile duct stones, or obvious tumor lesions in the biliary tract.

In light of these findings, further investigations into the cause of elevated hepatobiliary enzymes were conducted. Serological testing was negative for antinuclear antibodies and antimitochondrial antibodies, and the serum immunoglobulin G4 (IgG4) level was within the normal range. Based on these results, primary biliary cholangitis, autoimmune hepatitis, and IgG4-related disease were considered unlikely.

The patient’s clinical symptoms and hepatobiliary enzyme levels improved spontaneously, and he was discharged on hospital day 10. Two months later, he developed ileus and was readmitted following insertion of a decompression tube. Progressive stenosis of the ascending colon precluded lower gastrointestinal endoscopy. Although mucosal inflammation was observed, the mucosal architecture was preserved, with no evidence of epithelial neoplasia (Supplemental Digital Content Figure 1a, available at: https://links.lww.com/IJSCR/A119). Contrast-enhanced CT showed a persistent increase in fat attenuation around the duodenum, ascending colon, and anterior renal fascia, with diffuse small-bowel dilatation, a collapsed colon, and ascites (Supplemental Digital Content Figure 1b, available at: https://links.lww.com/IJSCR/A119). As the patient developed progressive bowel obstruction due to rapidly worsening colonic stenosis, and because conservative treatment was ineffective, surgical resection was performed for both diagnostic and therapeutic purposes. First, we performed a diagnostic laparoscopy to confirm the absence of obvious peritoneal seeding or tumors in the retroperitoneal organs. However, because we observed significant chronic inflammation and fibrosis in the retroperitoneum, we converted to open surgery and performed a right hemicolectomy.

**Figure 2.**
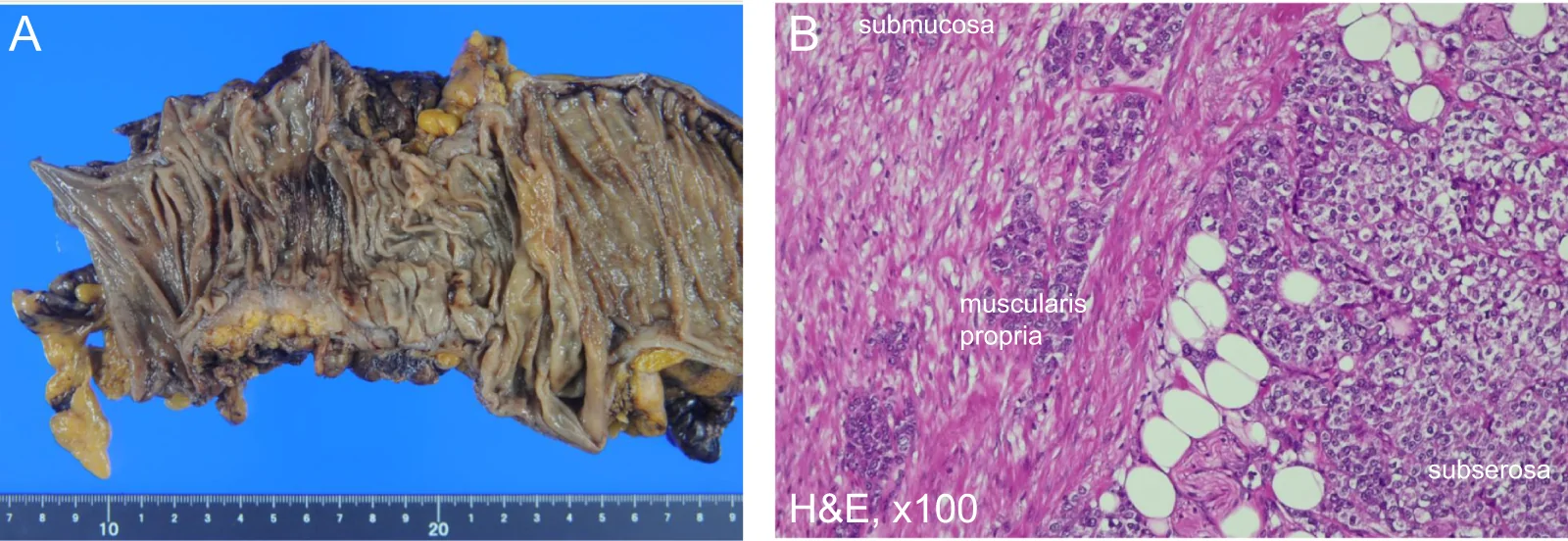
Histopathological findings of the resected ascending colon. (a) Gross appearance of the resected ascending colon, showing marked wall thickening and luminal narrowing. (b) Histopathological findings of the resected specimen, showing a poorly differentiated adenocarcinoma forming solid nests, infiltrating the colonic wall and extending from the muscularis propria into the subserosal adipose tissue (hematoxylin and eosin staining, ×100), consistent with metastatic carcinoma rather than a primary colorectal cancer.

Histopathological examination of the resected specimen revealed a poorly differentiated adenocarcinoma forming solid nests, proliferating within the colonic wall, and extending into the muscularis propria and subserosal adipose tissue. These findings indicated metastatic carcinoma involving the ascending colon (Fig. 2). Immunohistochemistry showed cytokeratin 7 positivity, with cytokeratin 20 and CDX2 negativity, arguing against primary colorectal cancer. Although focal thyroid transcription factor-1 (TTF-1) positivity suggested pulmonary adenocarcinoma, no elevation of tumor markers or pulmonary lesion was identified on imaging.

The patient was discharged on postoperative day 8 and followed up as an outpatient. One month postoperatively, PET-CT revealed a retroperitoneal mass located near the right renal hilum with an SUVmax of 6.5 (Supplemental Digital Content Figure 2a, b, available at: https://links.lww.com/IJSCR/A119). No abnormal FDG uptake was observed in the lungs or other organs, and the primary site remained unidentified. Three months postoperatively, the patient developed bowel obstruction and was readmitted. Contrast-enhanced CT showed progression of the right retroperitoneal mass (Supplemental Digital Content Figure 3a, available at: https://links.lww.com/IJSCR/A119). In addition, multiple hepatic and pulmonary metastases were detected, along with worsening right-sided hydronephrosis (Supplemental Digital Content Figure 3b, available at: https://links.lww.com/IJSCR/A119).

**Figure 3.**
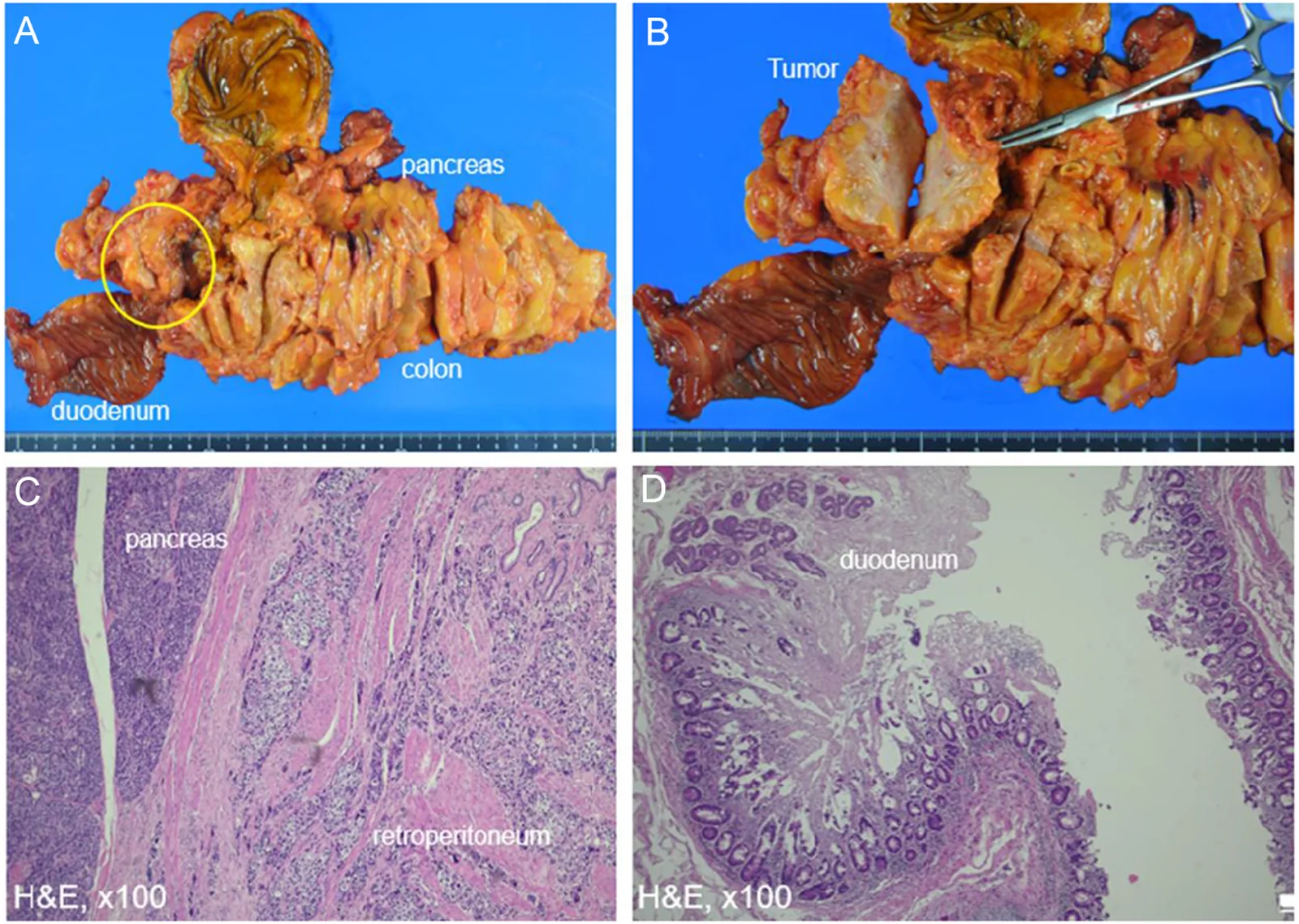
Pathological autopsy findings. (a) Gross appearance of the autopsy specimen shows a solid tumor located in the pancreatic groove, bordered by the pancreatic head, duodenum, and ascending colon (circled). (b) Cut surface of the tumor demonstrates a solid mass within the pancreatic groove. (c) Histopathological examination shows extensive direct invasion of the tumor into the retroperitoneum adjacent to the pancreas (hematoxylin and eosin staining). (d) Histopathological findings demonstrate direct invasion of the tumor into the duodenum (hematoxylin and eosin staining), supporting the pancreatic origin of the tumor.

The patient declined further chemotherapy and opted for supportive care. After resolution of the bowel obstruction had been confirmed by a contrast study through the ileal tube, the tube was removed. However, the bowel obstruction recurred shortly after the resumption of oral intake. Surgical intervention was performed, and a bypass between the small intestine and transverse colon was created. Subsequently, hydronephrosis caused by peritoneal dissemination led to rapid deterioration of renal function, necessitating placement of a right ureteral stent. The patient’s condition then deteriorated suddenly, and the patient was pronounced dead. The primary site of the malignancy was determined by pathological autopsy. The autopsy revealed a solid tumor measuring 5 × 5 × 4 cm located in the pancreatic groove, bordered by the pancreatic head, dorsal aspect of the duodenum, and common bile duct (Fig. 3a and b). In addition, multiple metastatic tumor nodules were identified in the liver and lungs. Histopathological examination demonstrated that the tumor arising in the pancreatic groove had directly and extensively invaded the retroperitoneum and duodenum (Fig. 3c and d). Furthermore, widespread tumor emboli were observed within the pulmonary arteries, which were considered to have contributed to the patient’s rapid clinical deterioration.

## Discussion

Groove pancreatic carcinoma arises within the anatomical space bordered by the pancreatic head, the descending portion of the duodenum, and the distal common bile duct – an area referred to as the “groove.” In contrast to conventional pancreatic head carcinomas, groove carcinomas demonstrate a distinct pattern of invasion and advance more slowly. Consequently, early-stage cases often lack definitive radiological or endoscopic findings, making the diagnosis particularly challenging[1].

Owing to its unique anatomical location, early clinical manifestations commonly include edema and circumferential narrowing of the duodenum[4]. Unlike conventional pancreatic head carcinoma, groove pancreatic carcinoma is not always associated with involvement of the main pancreatic duct or downstream dilation. Histologically, tumor cells may infiltrate the duodenal submucosa, causing luminal narrowing without any mucosal disruption or clear ductal involvement, complicating diagnosis. In the present case, involvement of the ascending colon and right ureter was considered to indicate direct retroperitoneal invasion, rather than peritoneal dissemination (Supplemental Digital Content Figure 4, available at: https://links.lww.com/IJSCR/A119). The retroperitoneal extension along the anatomical continuity surrounding the duodenum, right ureter, and ascending colon may explain the unusual invasion pattern. Moreover, serum tumor markers often remain within normal ranges, adding another layer of diagnostic difficulty[2]. Consistent with this challenge, imaging findings in groove-related pancreatic diseases often overlap with those of inflammatory and malignant conditions, making accurate preoperative differentiation difficult[5]. In the present case, focal TTF-1 positivity initially indicated metastatic pulmonary adenocarcinoma; however, no tumor marker elevation or imaging findings indicated lung cancer. Pathological autopsy findings supported the diagnosis of primary groove pancreatic carcinoma, with the tumor centered in the pancreatic groove and showing extensive direct invasion into the retroperitoneum and duodenum, without any evidence of a primary pulmonary lesion. Given that focal TTF-1 expression can occur in a subset of non-pulmonary adenocarcinomas, the overall anatomical distribution and invasion pattern were considered more consistent with a pancreatic origin than with metastatic lung cancer^[^2,6^]^. Colonic involvement in groove pancreatic carcinoma is extremely rare, with only two cases reported, and initial presentation with ascending colon stenosis is particularly uncommon^[^7,8^]^. Most reported cases present with symptoms of duodenal stenosis. Thus, the present case was unique in that ascending colon stenosis was an early manifestation preceding the diagnosis of groove pancreatic carcinoma[5].

In reported cases, pancreatoduodenectomy followed by adjuvant chemotherapy has been performed for groove pancreatic carcinoma, resulting in acceptable short-term outcomes despite its distinct growth pattern compared with that of conventional pancreatic ductal adenocarcinoma[9]. Although preoperative diagnosis remains challenging, repeated evaluation with ERCP and endoscopic ultrasound–guided fine-needle aspiration (EUS-FNA) may have enabled an earlier diagnosis. As endoscopic ultrasound and MRCP revealed no clear signs of malignancy, including the absence of an obvious pancreatic mass lesion, main pancreatic duct dilatation, or mucosal abnormalities, along with spontaneous improvement of hepatobiliary enzyme levels, we performed a right hemicolectomy to address both the diagnosis and treatment of the colonic stricture before pathological confirmation. Nevertheless, if groove pancreatic carcinoma had been diagnosed through repeated EUS-FNA, a favorable prognosis might have been achieved through multidisciplinary treatment. Earlier recognition may have enabled timely multidisciplinary treatment, including systemic chemotherapy and radical surgery[10].

## Conclusions

Herein, we report a case of rapidly progressive ascending colon stenosis with an aggressive clinical course. Groove pancreatic carcinoma should be considered in cases of unexplained colonic stenosis adjacent to the pancreatic groove, particularly in the presence of retroperitoneal inflammatory changes without obvious mucosal lesions or definitive pancreatic findings.

## Data Availability

The data supporting the findings of this case report are not publicly available due to concerns regarding patient privacy.
